# Comparative Study of Life Quality Between Migrant Children and Local Students in Small and Medium-Sized Cities in China

**DOI:** 10.1007/s10560-018-0545-5

**Published:** 2018-05-03

**Authors:** Junhua Zhang

**Affiliations:** 1 School of Education Science, Jiangsu Key Laboratory for Big Data of Psychology and Cognitive Science, Yancheng Teachers College, Yancheng Teachers University, 50 Kaifang Road, Yancheng, 224002 Jiangsu People’s Republic of China; 2 Department of Psychology, University of Southampton, Southampton, SO17 1BJ UK

**Keywords:** Migrant children, Migrant children, Quality of life, Social support, Small and medium-sized cities

## Abstract

Every child deserves a fair chance in life. However, migrant children are at higher risk of developing mental health problems. The problem of migrant children who have left their hukou registration place for 6 months or longer with their parents from rural areas to cities in China has become a unique social issue in the social transformation of China. However, even up to this day, little is known about life satisfaction of migrant children in small and medium-sized cities. To investigate the current situation of migrant children’s life satisfaction, several scales including Chinese Adolescent Students’ Life Satisfaction Questionnaire, Social Economic Status Scale, Social Support Rating Scale and big five inventory were used to obtain data on 142 migrant children and 165 local primary school students. Results showed that migrant children’s life satisfaction was significantly lower when compared to local non-migrant students. The study also highlighted that subjective and objective support, utilization of support, conscientiousness and parent’s educational level were predictive factors of life satisfaction. Migrant children’ life satisfaction was not optimistic and social support was significantly influencing factors of migrant children’s life satisfaction, so they need a support system of government, school, community, family to help them through difficulties.

Every child deserves a fair chance in life, however, migrant children are at higher risk of developing mental health problems (UNICEF, 2016) with significantly higher depression and disruptive behavior scores compared to their non-migrant counterparts (Kim et al., 2018). A change in the environment can have negative effects on migrant children, as it is harder for them to acclimate leaving these children often feeling lonely and misunderstood. To compensate, migrant children often work harder to acquire key skills such as communication skills and emotional resilience in order to integrate successfully with their new environment (Cankar, Deutsch, Dobrnjic, & Cankar, 2013). Alati found that length of stay was positively associated with aggression and delinquency amongst the children at both 5- and 14-years follow-up (Alati, Najman, Shuttlewood, Williams, & Bor, 2003). The change of living and learning environment can easily lead to psychological problems and social adjustment problems (Kim et al., 2018; St-Hilaire, 2002). A meta-analysis exploring the differences between migrant and native children’s mental health found that the influence of migration varied with the informants used and the characteristics of the migrant group (Stevens & Vollebergh, 2007). In America, Migrant Education Program (MEP) targeting migrant children resulting from changes in parental work has provided migrant children with high-quality services and received effectiveness (Bal & Perzegian, 2013). However, migrant children in China are those population under the age of 18 who have left their hukou registration place for 6 months or longer and most of them are migrating with workers from rural areas to cities (Wang, 2016). With the accelerating process of urbanization in China, more and more rural labor force has flowed into cities and now roughly 35.8 million migrant children move from original rural residence to urban areas with their parents (Lu et al., 2018). Since 2014, hukou policy has been gradually revised to allow more migrant children to receive public education in cities. However, even today, many civil benefits are still bound to hukou and many migrants still can’t enjoy the same social welfare benefits without local hukou because education and other social welfare of migrant children was not the responsibility of the destination city government according to the system of compulsory education funding. Many migrant children are still challenged by social exclusion and discrimination (Zhou & Cheung, 2017). The problem of migrant children in China has become a unique social issue in the social transformation of China and to understand their current situation is of great realistic and theoretical significance thanks to the particularity of household registration and the huge number.

From countryside to the city, the living and learning environment has changed a lot, many children have to leave their hometown and live in a narrow house. With less connection from original rural communities and less attention from a nearby parent, many of these migrant children are only slowly developing the socialization skills (Xiu-Yun, Xiao-Yi, Yang, & Jing, 2009). Because of long working hours, migrant parents usually have little time to communicate with their children, even though they desire to have more interaction with their kids (Feng, 2015). At the same time, migrant children had to turn to privately-operated migrant schools, which are usually under-funded, have bad environmental facilities and lack adequate teaching compared to public schools (Liu, Zhou, & Feng, 2016). It was found that rural migrant students were exposed to more risks but are not more delinquency-prone than non-migrant students (Liu & Liu, 2016). With the social concern about this issue, the enrollment of migrant children has been greatly improved and the focus has gradually expanded from educational opportunities to all aspects of migrant children’s development (Hao & Shan, 2011). After getting the opportunity to study in the city, the next important problem is how well they adapt.

Life quality focuses on excellence or goodness in aspects of life that go beyond mere subsistence, survival, and longevity. It is not only an important parameter of personal subjective well-being based on his own criteria but also an important index of social adaptation (Lang & Hellweg, 2006). Educational and recreational resources are objective indicators of life quality. But in consider of cost and convince, subjective measures of life quality are used commonly, which refers to “a person’s subjective evaluation of the degree to which his or her most important needs, goals, and wishes have been fulfilled, an overall or global evaluation of quality of life as well as evaluations of species life domains life satisfaction (Huebner, Suldo, Smith, & McKnight, 2004)”. It has been found that, compared with other demographic variables, family socio economic status (SES) has a greater impact on individual well-being (Choi, Kim, & Park, 2016). Members of lower SES are more likely to have bad health outcomes (Dalstra et al., 2005). Personality is one of the most reliable and potent indicators of well-being (Chen, Gao, & Shen, 2012). The profiles of personality traits of well-being and unfortunate people are very different (DeNeve & Cooper, 1998). Social support is also an important factor affecting life satisfaction and those who receive various kinds of social support feel higher levels of well-being (Kolarcik, Geckova, Reijneveld, & van Dijk, 2012). Migrant children’s mental health is decreased by rural-to-urban migration (Wang, Liu, Zheng, Liu, & You, 2017). Due to the lack of adult supervision, migrant children after school either watch TV at home or shuttle around the school, streets, shops, internet cafes and game rooms. On the one hand, they will be discriminated by urban people and vulnerable to the temptation of violent pornography, which will affect the social adaptation, because perceived discrimination is hypothesized to influence various outcomes (Sanchez & Brock, 1996). Migrant children are disadvantaged by the sociocultural circumstances in urban areas (Sun, Chen, & Chan, 2016).

Compared with those mega-cities such as Beijing, Shanghai, and Guangzhou, small and medium-sized cities have less pressure of population and have gradually revised their hukou policies (local household registration) which allow migrant children to receive public education in cities and integrates them into the mainstream educational system (Zhou & Cheung, 2017). These entitlements will encourage increasing numbers of migrant children to enter in small and medium-sized cities whose urban resident population is less than 1,000,000 persons. Nowadays, small and medium-sized cities played more and more important roles in China’s new urbanization strategies (Lv & Destech Publications Inc., 2015; Meng et al., 2010), the government began to emphasize the necessity of coordinating the development of small, medium and large cities. Because rural-to-urban migration is a potential risk factor for the mental health of migrant children (Wang et al., 2017), life satisfaction of migrant children after entering schools in small and medium-sized cities is still unclear, which is critical both to the healthy growth of migrant children and the stability of the entire nation as a whole. In this context, this study focused on the comparison of life satisfaction among migrant children and local students in small and medium-sized cities in China. In sum, we had two hypotheses.

1. Life satisfaction of migrant children will be lower than that of local students.
2. Family SES, social support, and personality have a significant influence on the life satisfaction of this special group.

## Methods

### Respondents

The study was conducted in two primary schools in the city of Changshu situated in the Jiangsu province, China. Changshu ranked fourth in the 2017 annual comprehensive strength of small and medium cities in China. Ethical approval was granted by the Institutional Review Board (IRB) of the Yancheng Teachers University. A questionnaire was disseminated and completed anonymously by 142 migrant children (86 males and 56 females) and 165 local students (73 males and 92 females) taking part in this study. To obtain consent a letter was sent to parents 10 days before the study began. The letter comprised a brief description of the study, informed parents their right to withdraw, and assured all data is confidential. Implied consent was acquired through completion of the questionnaire and if parents did not refuse their child’s involvement in the study. Children granted permission by their parents were re-informed their participation in the study was entirely voluntary.

### Instrumentation and Measures

Life satisfaction was measured by Chinese Adolescent Students’ Life Satisfaction Questionnaire. The questionnaire consists of 36 questions split into six satisfaction subscales: friendship, family, school, academic, freedom and environmental. Questions included in the questionnaire It used a 7-point scale (from strongly agree to strongly disagree). Questions items include “I can do what I like to do in my spare time” etc. The reliability of the total scale was 0.93, and that of the subscale was between 0.76 and 0.87. Exploratory factor analysis showed that cumulative variance of six factors was 49.154%, indicating that the scale had good structural validity two (Zhang, He, & Zheng, 2004).

The SES questionnaire was compiled with reference to the sociological theory. The main indicators are the educational level of parents and their parents’ job scores at 5-point scale. For example, the education level of your father is (1) ≤ elementary school, (2) junior high school, (3) high school, (4) university, (5) ≥ graduate. The higher score means the higher SES. The internal consistency of reliability in this survey was 0.79 (Shi & Shen, 2007).

Social Support Rating Scale (SSRS) has three dimensions and they are objective support, subjective support, and social support. A higher score indicates that one gets more social support. Considered the actual situation of students, some of the items in the scale were revised. “Colleagues” are changed to “classmates” and “spouse”, “children” are changed into “friends”. Reliability of the total scale was found to be 0.92 (Xiao, 1994). Items are just like that.

Big five inventory (BFI) contains five facets of neuroticism, extraversion, openness, agreeableness, and conscientiousness. This scale has good reliability. The higher the score is in extraversion, openness, agreeableness, and conscientiousness, the better adaption is (Hellriegel, Slocum, & Woodman, 2001).

### Data Analysis

The data were statistically analyzed using Excel and SPSS statistical software. Descriptive statistical analysis, *t* test, and stepwise regression analysis, was calculated.* p* < 0.05 for the difference was statistically significant.

## Results

### Compaction of Life Satisfaction Among Migrant Children and Local Students

As shown in Table 1, migrant children’ total life satisfaction, friendship satisfaction, family satisfaction, academic satisfaction, freedom satisfaction, and environment satisfaction, school satisfaction were significantly lower than those of local students. As seen in Table 2, migrant children and local students had a significant difference in the family SES and social support. Migrant children’ SES were significantly lower than their urban local companions. Among the five facets of personality, the scores of conscientiousness and openness among migrant children were also significantly lower.

**Table 1:** Life quality score between migrant children and local students

	Local students (n = 165)	MIGRANT children (n = 142)	*t*
Friendship satisfaction	5.63 ± 0.96	4.87 ± 0.97	6.87***
Family satisfaction	6.20 ± 0.87	5.53 ± 1.09	5.87***
Academic satisfaction	4.80 ± 1.14	4.11 ± 0.94	5.78***
Freedom satisfaction	5.39 ± 1.18	4.65 ± 1.13	5.61***
School satisfaction	5.86 ± 1.08	5.02 ± 1.13	6.70***
Environment satisfaction	5.68 ± 1.02	4.85 ± 1.02	7.05***

^***^p < 0.001

**Table 2:** Relative factors between migrant children and local students

	Local students	Migrant children	*t*
Education of father	3.44 ± 0.92	2.58 ± 0.64	9.57***
Education of mother	3.48 ± 0.87	2.29 ± 0.79	12.52***
Occupation of father	3.07 ± 1.08	2.26 ± 0.65	8.03***
Occupation of mother	2.82 ± 1.00	2.06 ± 0.82	7.38***
Subjective support	4.04 ± 0.86	3.35 ± 0.85	7.15***
Objective support	4.11 ± 0.74	3.64 ± 0.81	5.29***
Utilization of support	3.96 ± 0.90	3.44 ± 0.88	5.11***
Neuroticism	45.54 ± 11.43	46.69 ± 11.35	− 0.86
Extraversion	52.98 ± 8.13	52.18 ± 9.12	0.81
Openness	53.61 ± 8.73	51.00 ± 9.70	2.48*
Agreeableness	53.29 ± 11.78	51.81 ± 12.20	1.08
Conscientiousness	54.71 ± 8.79	51.43 ± 8.21	3.36***

^***^p < 0.001; ^*^p < 0.05

### Correlation Analysis and Regression Analysis

As shown in Table 3, there were significant correlations between dimensions of life satisfaction, SES, social support, and personality. In order to compare the relative contribution of the life satisfaction, Stepwise logistic regression was performed using facets of SES, social support and personality as the dependent variable, and life satisfaction as the independent variable. As shown in Table 4, subjective support, conscientiousness, objective support, father’s education level, mother’s occupation and utilization of support had significant predictive effects on migrant children’ life satisfaction, among which subjective support had the strongest predominance.

**Table 3:** Correlation between life quality and relative factors among migrant children

	Friendship satisfaction	Family satisfaction	Academic satisfaction	Freedom satisfaction	School satisfaction	Environment satisfaction
Education of father	0.31^***^	0.30^***^	0.28^***^	0.27^***^	0.21^***^	0.31^***^
Education of mother	0.30^***^	0.32^***^	0.28^***^	0.26^***^	0.27^***^	0.33^***^
Occupation of father	0.24^***^	0.18^**^	0.22^***^	0.15^**^	0.16^**^	0.29^***^
Occupation of mother	0.25^***^	0.18^**^	0.21^***^	0.13^*^	0.21^***^	0.28^***^
Subjective support	0.67^***^	0.47^***^	0.53^***^	0.43^***^	0.45^***^	0.44^***^
Objective support	0.47^***^	0.51^***^	0.41^***^	0.39^***^	0.38^***^	0.37^***^
Utilization of support	0.48^***^	0.47^***^	0.36^***^	0.31^***^	0.39^***^	0.35^***^
Neuroticism	− 0.13^*^	− 0.12^*^	− 0.18^**^	− 0.14^*^	− 0.13^*^	− 0.17^**^
Extraversion	0.26^***^	0.19^**^	0.16^**^	0.18^**^	0.20^**^	0.26^***^
Openness	0.18^**^	0.07	0.13^*^	0.19^**^	0.04	0.13^*^
Agreeableness	0.31^***^	0.22^***^	0.28^***^	0.22^***^	0.26^***^	0.35^***^
Conscientiousness	0.36^***^	0.26^***^	0.38^***^	0.33^***^	0.26^***^	0.31^***^

^***^p < 0.001; ^**^p < 0.01; ^*^p < 0.05

**Table 4:** Stepwise regression analysis of life quality among migrant children

	*Β*	*β*	*t*	*R* ^2^	*F*
				0.524	54.482^***^
Subjective support	1.967	0.355	6.143^***^		
Conscientiousness	0.098	0.167	3.815^***^		
Objective support	1.161	0.183	3.275^**^		
Education of father	0.709	0.126	2.828^**^		
Occupation of mother	0.615	0.119	2.747^**^		
Utilization of support	0.604	0.109	1.971^*^		

^***^p < 0.001; ^**^p < 0.01; ^*^p < 0.05

## Discussion

### Inferior Life Satisfaction of Migrant Children

The scores of total life satisfaction, life satisfaction, friendship satisfaction, family satisfaction, academic satisfaction, freedom satisfaction, environmental satisfaction and school satisfaction were above 4 in the 7 point Likert scales. So the overall life Satisfaction of migrant children was not too bad. But the scores of migrant children were significantly lower than that of local students, especially environment satisfaction which was much lower deserved more attention. The current situation of migrant children is not optimistic and their life quality is lower than their urban counterparts. The results are similar to the previous study on the migrant children in big cities (Sun et al., 2016). Compared to the urban children, Migrant children showed more problem behaviors especially the internalizing problem and their self-concept level was significantly lower (Li, Zou, Jin, Can, & Ke, 2008). In the interaction with teachers and peers, they deliberately tended to keep a certain distance and were often not confident (Wang, Lin, Hou, & Fang, 2016).

### Internal and External Factors Affecting Life Satisfaction of Migrant Children

The education and occupations of migrant children’s parents scored significantly lower than their urban companions. The lower status of migrant workers was in line with the actual situation because the vast majority of migrant workers were not well educated and professional. While the father’s education level and mother’s occupation status had significant predictive effects on the life satisfaction of migrant children. High education level parents usually spent more time and money on children’s education, and respected children more and used less simple and crude mode of education, while a mother with low occupation status had to work for a long time and had not enough time and mood to accompany and educate her children.

Migrant children scored significantly lower than that of local students in the facets of openness and conscientiousness. Openness here included interpersonal openness as well as an exploration of new things, which may be related to migrant children’s psychological stimulus and psychological fall from rural to urban areas. If one only likes familiar things and dislike new things, he will be more easily introverted (Zhang, 2010). Conscientiousness refers to self-discipline and self-control. The low conscientiousness of migrant children was mainly due to the civilized leap from rural areas to urban areas. Original set of “wild” living habits in rural areas did not adapt to urban life and migrant children encountered great resistance and challenges in integrating with the new reference system and evaluation system. Their urban life experiences and rules need to be expanded, and their planning, consistency, and persistence of doing things also need to be improved urgently. Conscientiousness had a significant predictive effect on the life satisfaction of migrant children, which was due to the fact that familiarity can bring familiarizes and sense of security, which can lead to satisfaction. Therefore, norms and rules of living should be given to urban migrant children to enable them to know how to make rational use of urban living facilities and equipment so that they can quickly integrate into urban life.

It was found that social support among migrant children scored significantly lower than that of local students. The results were related to the existing hukou system and resource allocation system which has caused a serious breakdown between urban and rural areas in China (Sun, 2004). All three factors of social support could significantly predict the life satisfaction of migrant children, which proved once again that social support played an important role in promoting their life satisfaction. The higher the level of social support, the higher the life satisfaction of migrant children was. Reasons lied in the fact that migrant students who got more social support usually were more confident and calmer-to cope with various difficulties with learning and social life, which led to higher life satisfaction. Since the social support of migrant children had a positive effect on the improvement of their life satisfaction, it can be inferred that to some extent the pilot of trans-provincial cooperative medical care, compulsory education and other initiatives would improve the life satisfaction of migrant children in the future.

The importance of subjective, objective support, utilization of support also reconfirm the previous research about rural children (Ye, Shen, & Qiu, 2017). Among three subscales of social support, subjective support had highest predictive power for life satisfaction of migrant children, which was consistent with the results of Zheng who found that subjective support played an important part in subjective well-being (Zheng & Chen, 2005). Therefore, in addition to providing practical social support for migrant children, Training in comprehension and gratitude is also needed so that migrant child will be more willing to accept various kinds of support from schools, peer friends, and the whole society, and enhance their expectations and evaluations of social support. Compassion is not supporting and Support should be given correctly and efficiently. Otherwise, it will be very difficult to improve their life satisfaction and integrates them into the mainstream urban system.

## Practical Implication

Migrant children’ life satisfaction was significantly lower than that of local peers, so they need a support system of government, school, community, family to help them through difficulties. It is suggested that government should adopt educational policies to address their needs and barriers and deliver affordable psychological services to millions of migrant children. Therefore conditional provinces and cities to take more active measures to solve invisible discrimination (Wang & Jiang, 2016). Make good use of big data to reduce the cost and achieve tangible results. Personalized guidance and service should be offered to potential needier. Schools and teachers should master more practical and simple-to-use educational methods and increase the communication chance between migrant children and naive children, such as sand play games and group counseling, in order to eliminate discrimination and enhance migrant children’s sense of belonging and social integration (Zhuang, 2013). Parents are important resources and parental discriminatory understanding can have significant implications for migrant children’s adjustment (Hou, Kim, Hazen, & Benner, 2017). Parents desperately need to change the educational idea, turn more attention from child’s academic scores into children’s mood, and narrow the gap between what their children want and what parents can offer.

Communities in cities should create a fair and orderly social atmosphere, give respect and equal opportunities to migrant children. And most importantly, migrant children should go within themselves, learn to get more value from limited support and make thing better through self-education because of scarce external resources. For example, spirituality may have the potential to answer migrant children’ deep existential needs, and constitute a key resilience factor (Vinueza, 2017).

## Limitations and Suggestions for Further Studies

The first limitation was about the measurement instruments. First, the study used self-reported data, which limited the perspective of China. Another objective index should be included. Another limitation of measurement involved the missing value of students, which may lead to representation problem. The third limitation is that some instruments used were made by Chinese and there was no worldwide validation. In the future study, more worldwide instruments and more objective index should be included. Factors that may influence the results, such as emotional intelligence and parenting style were not included. The reason is that the focus of this study was on easily measured and identifiable factors, as too many items would only make children bored.
